# Microbial Interactions with Intestinal Lipid Digestion and Absorption: Emerging Targets for Metabolic Disorders

**DOI:** 10.34133/research.0904

**Published:** 2025-09-19

**Authors:** Jie Yin, Mingliang Zhang, Weimin Jiang, Xingguo Huang, Yulong Yin

**Affiliations:** ^1^ College of Animal Science and Technology, Hunan Agricultural University, Changsha 410128, China.; ^2^ Hunan Institute of Animal and Veterinary Science, Changsha 410131, China.; ^3^ Institute of Subtropical Agriculture, Chinese Academy of Sciences, Changsha 410125, China.

## Abstract

Dietary fat undergoes digestion and absorption before entering enterocytes, where lipids are re-esterified and packaged into chylomicrons for lymphatic transport. The interaction between intestinal lipid absorption and metabolic disorders, such as obesity, has been demonstrated in both animal models and human studies. Here, we comprehensively review microbial interactions with intestinal lipase secretion and lipid absorption as well as the potential mechanisms associated with microbial metabolites, bile acids, immune cells, Snhg9, toll-like receptors, and intestinal permeability. We also highlight the relevance of these findings to metabolic diseases and their potential application in developing therapies that target intestinal lipid absorption through the modulation of gut microbiota, such as the use of probiotics and dietary nutrients. Finally, key questions regarding microbial interactions with intestinal lipid absorption are outlined to guide future research.

## Introduction

Diets provide a variety of nutrients (i.e., amino acids, carbohydrates, lipids, vitamins, and minerals) for the survival and growth of animals and humans, which are absorbed into the body across the gastrointestinal tract. An appropriate intake of dietary fats, including triglycerides (TGs), cholesterol esters, and phospholipids, is critical for maintaining human health by regulating satiety and energy homeostasis [1]. The adherence to high-fat foods along with improved living standards that exceeds the current average level of fat requirement results in excess absorption and deposition of lipids, ultimately leading to body weight gain and high occurrence of metabolic diseases, such as obesity [2], arteriosclerosis [3], fatty liver diseases [4], and diabetes [5]. In particular, a dietary pattern rich in high energy and saturated fat intake promotes overweight or obesity by accelerating lipid absorption [6]; nevertheless, a low saturated fat intake for 2 years improves insulin sensitivity in children with a family history of obesity [7]. Thus, governing intestinal lipid absorption is crucial for the effective treatment and prevention of diseases associated with lipid metabolic disorders, which contribute markedly to morbidity and mortality.

Unlike that for other macronutrients transported into intestinal capillaries, the absorption pathway of lipids is mainly through the gastrointestinal lymphatic system [8]. Briefly, the ingested TG is hydrolyzed into monoglycerides and fatty acids with the assistance of lipase; then, the lipids are emulsified by bile acids to form micelles containing monoglycerides, fatty acids, cholesterol, and phospholipids [9]. Gut lipid-transporting systems (i.e., CD36, fatty-acid-binding proteins [FABPs], fatty acid transport proteins, and Niemann–Pick C1 like-1 [NPC1L1]) further help to transfer the micelles into enterocytes, where lipids are re-esterified into TGs and subsequently assembled into chylomicrons along with proteins, phospholipids, and cholesterol to enter the intestinal lymphatics [9]. In recent years, numerous high-quality reviews have been published on intestinal lipid absorption, with particular focus on lipid transport proteins that mediate lipid absorption and help prevent metabolic diseases [8,10,11].

Dietary fat is primarily digested and absorbed in the small intestine, which contains a dense and diverse population of microbial cells [12]. Antibiotic exposure, germ-free animals, and fecal microbiota transplantation (FMT) have widely confirmed the role of gut microbiota in lipid digestion and absorption [13–15]. Thus, modulation of gut microbiota to target intestinal lipid absorption may offer therapeutic potential for metabolic diseases through probiotic or diet-based interventions.

## Gut Microbiota and Fat Digestion

**Fig. 1.**
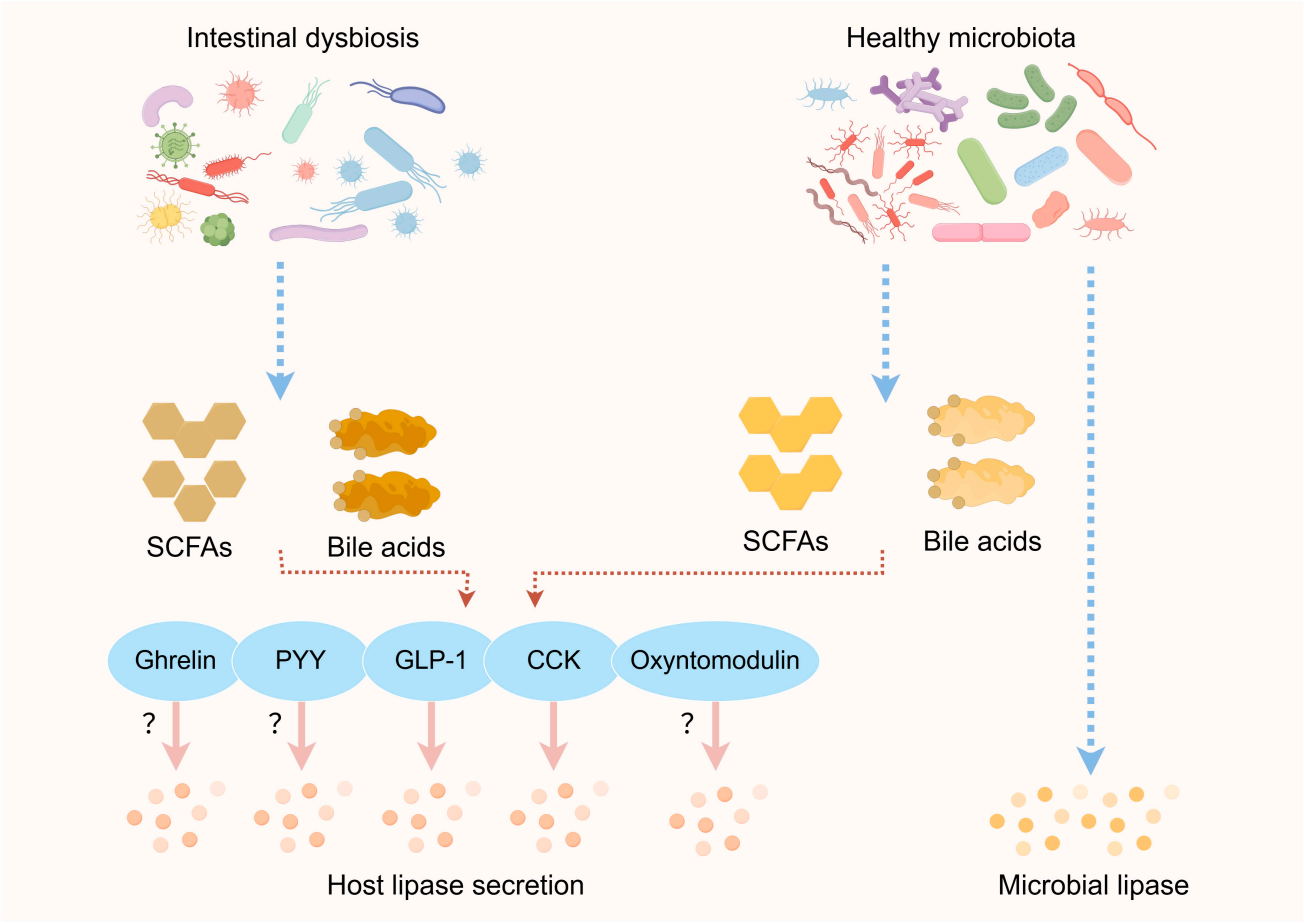
Gut microbiota is involved in fat digestion: (a) microbiota stimulates lipase secretion by pancreas and (b) microbial lipase helps fat hydrolysis into fatty acids. SCFAs, short-chain fatty acids; PYY, peptide YY; GLP-1, glucagon-like peptide-1; CCK, cholecystokinin.

Germ-free mice exhibit markedly lower plasma TG levels and a higher fecal TG content, accompanied by an anti-obesity phenotype [14], suggesting a role for gut microbiota in regulating fat digestion capacity [16]. Notably, dietary TG cannot be directly absorbed by intestinal epithelial cells and must first be hydrolyzed into fatty acids through lipase-mediated digestion. Gut microbiota is involved in fat digestion in 2 independent pathways: (a) microbiota stimulates lipase secretion by pancreas and (b) microbial lipase helps fat hydrolysis into fatty acids (Fig. 1).

### Gut microbiota and host lipase secretion

The model in which cholecystokinin (CCK) evokes pancreatic lipase secretion is a well-established mechanism of satiation, primarily in response to dietary fat [17,18]. Healthy intestinal microbiota appears to promote CCK-dependent lipase secretion, as evidenced by the observation that antibiotic-induced dysbiosis alleviates CCK overexpression in the ileum of a mouse model of moderate fructose malabsorption [19]. Indeed, specific pathogen-free mice exhibit up-regulated *CCK* expression in the duodenum, jejunum, and pancreas, accompanied by increased lipase secretion during a lipid tolerance test using corn oil gavage, whereas germ-free mice fail to show an elevated CCK response [14]. The mechanisms may involve microbial metabolites, as exposure of enteroendocrine cells to heat-killed bacteria leads to increased CCK secretion [20]. In particular, bacteria-produced propionate activates the expression of *CCK* in different models [19]. However, the extent of CCK secretion is most pronounced in response to pathogenic bacteria, whereas it is least pronounced in response to nonpathogenic microbiota [20].

Glucagon-like peptide-1 (GLP-1) serves as another key target of microbial interaction with pancreatic lipase secretion. Depletion of the gut microbiome, induced by antibiotic exposure or in germ-free models, completely abolishes the postprandial GLP-1 response in both the circulation and ileum; this response is restored following gut microbial reconstitution via FMT [21]. These findings suggest that a healthy gut microbiota is essential for sustaining the postprandial GLP-1 response. Several bacteria are identified to activate GLP-1 secretion, such as *Akkermansia muciniphila* and *Parabacteroides distasonis* [22,23]. Currently, gut-microbiota-produced proteins interacting with intercellular adhesion molecule 2 [22] and dipeptidyl peptidase 4 [24], as well as interactions between the metabolites bile acids and Takeda G protein-coupled receptor 5 (TGR5) signals [21,23], are recognized to participate in the regulation of postprandial GLP-1 response to initiate lipase secretion. Notably, GLP-1 does not directly stimulate the secretion of amylase or lipase. However, GLP-1 induces the phosphorylation of the epidermal growth factor receptor and activation of Foxo1, thereby promoting acinar cell growth and the secretion of associated enzymes [25]. Interestingly, this effect appears to be mediated by GLP-1-induced redistribution of c-Src from the perinuclear region to the cell periphery [25].

Moreover, gut hormones, such as peptide YY, ghrelin, and oxyntomodulin, are also influenced by gut microbiota to participate in nutrient intake and metabolism [26–29]; further, direct mechanisms of gut microbiota–hormone signals on pancreatic lipase secretion for host metabolic manipulation need to be studied to provide novel insights into the interaction between gut microbiota and lipid absorption.

### Microbial lipase

The intestine hosts a vast number of microbial cells that not only stimulate the host’s lipase secretion but also directly produce lipases to facilitate dietary fat digestion. Since the first discovery of microbial lipases from *Aspergillus flavus* and *Penicillium oxalicum* in 1935, more than 4,700 bacterial lipase protein sequences have been identified, primarily from genera such as *Bacillus*, *Staphylococcus*, *Arthrobacter*, *Achromobacter*, and *Pseudomonas* [30]. Furthermore, lipase sequences in archaea and fungi exhibit homology with those in bacteria [30]. Presently, microbial lipases are attracting significant interest owing to their advantages of pH adaptability and heat stability [31]. Specifically, microbial lipases exhibit high enzymatic activity and can efficiently hydrolyze dietary fats in both acidic and mildly alkaline environments of the intestine. The resulting fatty acids are more readily absorbed by intestinal epithelial cells. Notably, the secretion and activity of microbial lipases are regulated by various factors, including intestinal pH, nutrient types, and their concentrations.

Current studies mainly focus on microbial sources, genome editing technology, purification techniques, and industrial applications, and more detailed discussion can be found in previous reviews [32,33]. Although gut microbiota plays a role in the production of intestinal lipases, the mechanisms by which microbiota-derived lipase lipid digestion and absorption contribute to metabolic diseases remain unclear. Therefore, future research should further explore the mechanisms underlying the interaction between gut microbiota and the host, particularly how microbiota influences host fat metabolism and energy balance through the secretion of lipase. This can be addressed using multi-omics approaches, including genomics, proteomics, and metabolomics. Additionally, real-time monitoring technologies—such as fluorescence labeling and biosensors—can be developed to track dynamic changes in microbial lipase activity within the intestinal tract. These tools will facilitate a deeper understanding of the mechanisms by which microbial lipase functions in the host.

## Gut Microbiota and Lipid Absorption

**Fig. 2.**
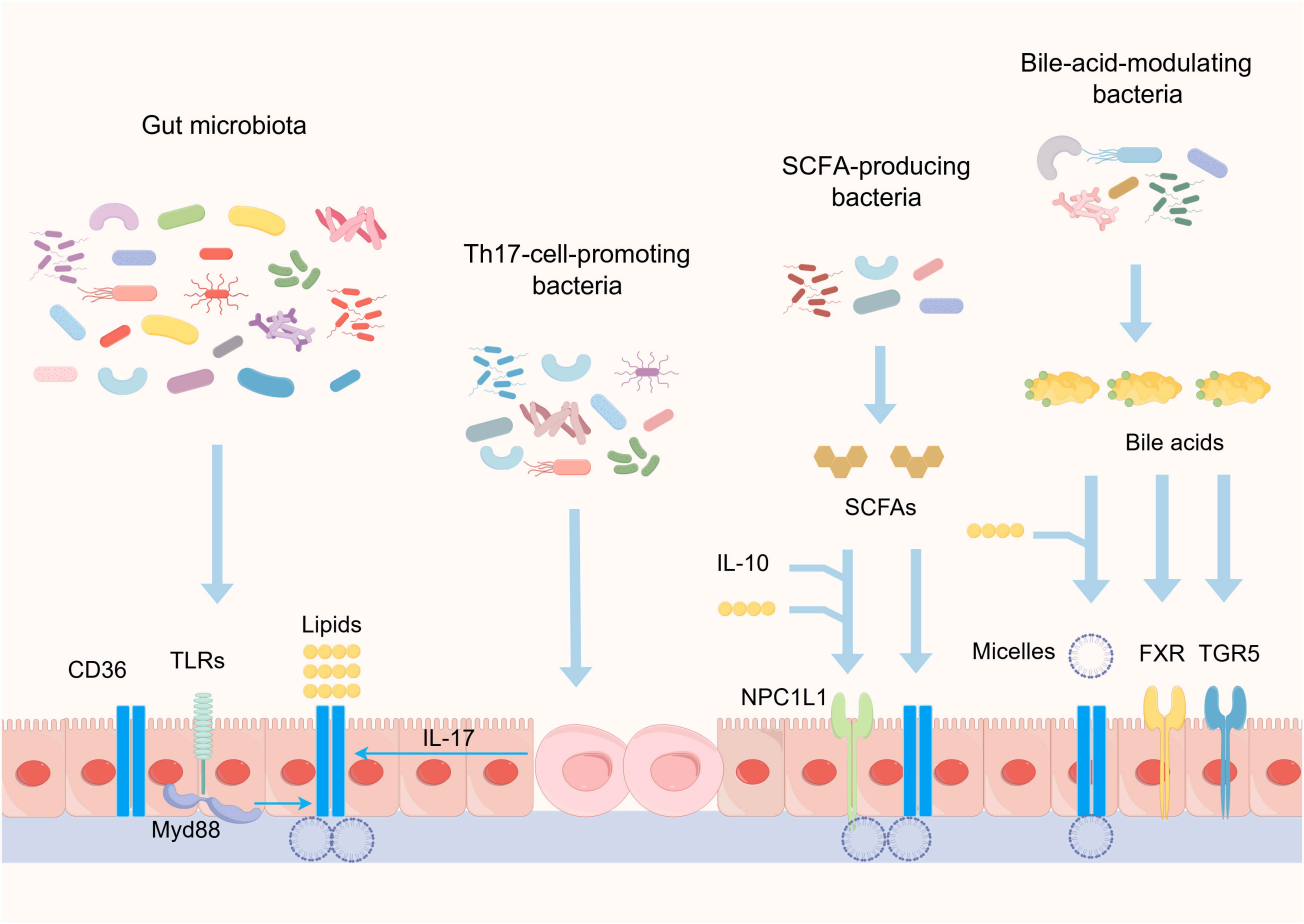
Gut microbiota and lipid absorption through microbial metabolites, bile acids, and immune cells. Firstly, healthy microbiota inactivates toll-like receptor (TLR)–Myd88 signaling to inhibit CD36-mediated lipid absorption. In addition, various bacteria display T helper 17 (Th17)-cell-promoting, SCFA-producing, and bile-acid-modulating effects, which further interact with lipid absorption through the interleukin-17 (IL-17), SCFA, and bile acid signaling pathways. IL-10, interleukin-10; NPC1L1, Niemann–Pick C1 like-1; FXR, farnesoid X receptor; TGR5, Takeda G protein-coupled receptor 5.

The direct evidence of gut-microbiota-mediated lipid absorption comes from germ-free mice, which exhibit a reduced rate of radiolabeled lipid absorption and deposition in the duodenum, jejunum, and liver [14]. Notably, antibiotic exposure also reduces mucosal apolipoprotein levels and impairs the lymphatic transport of TG and phospholipids [15]. To investigate whether microbial reconstruction can enhance intestinal lipid absorption, Martinez-Guryn et al. [14] transferred jejunal microbiota from high-fat-diet-fed mice to germ-free animals and observed restored lipid absorption capacity. Notably, the intestinal microbiota participates in nutrient digestion and metabolism and indirectly influences host nutrient absorption [34]. Clostridiaceae, *Lactobacillus* [14], Desulfovibrionales [35], and *Bifidobacterium* [36] have been reported to affect intestinal lipid absorption by targeting microbial metabolites, bile acids, and immune cells (Fig. 2). Furthermore, emerging evidence indicates that the interaction between the gut microbiome and intestinal permeability influences host lipid metabolism, suggesting that targeting intestinal permeability may also offer a therapeutic strategy for lipid metabolism disorders [37].

### Interacting with lipid transporters by microbial metabolites

Obesity-promoting bacteria are commonly reported to influence intestinal lipid digestion and absorption. For example, *Bacteroides thetaiotaomicron* exposure accelerates high-fat-diet-induced increases in body weight gain, glucose intolerance, fasting glucose level, and liver accumulation of fatty acids, which may be associated with the up-regulations of intestinal *CD36* and *FABP2*, 2 key lipid transporters [38]. Interestingly, some studies have reported that the abundance of *B. thetaiotaomicron* is reduced in obese individuals and that it alleviates diet-induced weight gain and obesity [39]. This variability may be attributed to genetic variations and metabolic differences among *B. thetaiotaomicron* strains, as well as to the host’s specific dietary background and the intestinal microecological environment. Furthermore, altered *Clostridia* abundance is a characteristic feature of metabolic syndrome and obesity. In germ-free animals, colonization with live *Clostridia* or exposure of intestinal epithelial cells to *Clostridia*-derived supernatants inhibits the expression of lipid-absorption-related genes and reduces lipid deposition, indicating a role for microbial metabolites [40].

Gut-microbiota-derived short-chain fatty acids (SCFAs), including acetate, propionate, and butyrate, are well-studied mediators of host–microbe metabolic interactions [41,42]. With the development of whole-genome sequencing, metabolomics, and culturomics, SCFA-producing bacteria are widely isolated and the biochemical process of SCFA release and influencing factors are fully determined [43,44]. Oral administration with an SCFA mixture has been reported to down-regulate *CD36* [45], a key fatty acid transporter, which functions in fat sensing and transport to govern lipid metabolism [10]. Specifically, propionate suppresses intestinal cholesterol absorption by targeting the NPC1L1 transporter in both humans and animals. This effect may be mediated by increased levels of interleukin-10, as inhibition of interleukin-10 receptor signaling abolishes propionate’s beneficial effects on total and low-density lipoprotein cholesterol and exacerbates atherosclerotic lesion severity [46]. In Caco-2 cells, butyrate, but not acetate and propionate, also reduces the expression of *NPC1L1*, concomitant with improvements in low-density lipoprotein cholesterol and atherosclerotic lesion area [47].

Recently, gut microbial degradation of myoinositol has been identified as a promoter of lipid absorption and obesity by targeting intestinal lipid transporters [48]. Myoinositol comes from various foods and bacterial metabolism of phytate. Intestinal myoinositol tends to inhibit intestinal lipid uptake by down-regulating fatty acid transport proteins, including CD36 and FABP2, which is in contrast to the observations in mice colonized by bacteria encoding the enzyme in the myoinositol degradation pathway, such as the genus *Megamonas*. Indeed, *Megamonas* is the most markedly elevated genus in obesity, and obese individuals carrying *Megamonas* show higher body mass index, body weight, waist circumference, hip circumference, 2-h postprandial glucose, and 2-h postprandial plasma insulin levels [48]. Thus, enhancing myoinositol-producing bacteria and reducing the genus *Megamonas* to manipulate intestinal myoinositol degradation are suggested to govern healthy lipid transport.

### Interacting with bile acids

The interaction between gut microbiota and bile acids is well-documented to facilitate intestinal lipid absorption, thereby modulating host metabolism [49,50]. Primarily, gut microbiota participates in the de novo conversion of cholesterol into cholic acid and chenodeoxycholic acid, which are subsequently conjugated with taurine or glycine and secreted into the intestinal lumen to emulsify lipids. Germ-free animals exhibit distinct bile acid profiles, characterized by elevated levels in the gallbladder and small intestine and reduced levels in the cecum, colon, feces, and serum, partially due to overexpression of genes involved in bile acid synthesis [51]. Supplementation with several probiotics, such as *Lactobacillus rhamnosus*, decreases de novo bile acid synthesis and enhances bile acid excretion, concomitant with improvements in liver injury and fibrosis in mice [52].

Bile acid modulation is a well-established mechanism by which gut microbiota interacts with host metabolism [53,54]. Due to the detergent properties, most intestinal microbiota has evolved resistance to conjugated bile acids by producing bile salt hydrolase, which performs deconjugation and biotransformations, yielding secondary bile acids such as hyodeoxycholic acid (HDCA), deoxycholic acid (DCA), ursodeoxycholic acid, and lithocholic acid (LCA) [55–57]. Predictively, mice with antibiotic exposure display a decreased profile of secondary bile acids [58]. The metabolic profiles of gut-microbiota-modified bile acids are generally altered in lipid metabolic disorders. For example, HDCA levels are reduced in the serum, liver, and intestine of patients with nonalcoholic fatty liver disease and the serum content is inversely correlated with the severity [59,60]. FMT from dyslipidemic donors also results in a reduced intestinal HDCA content accompanying increased lipid absorption [61].

Farnesoid X receptor (FXR) serves as a master regulator of bile acid metabolism, and activation by its agonist GSK2324 results in decreased lipid absorption, consistent with reduced plasma fatty acid and TG levels [4]. In addition, FXR-mediated reduction in lipid absorption is reversed after bile acid exposure, suggesting that FXR-dependent intestinal lipid absorption is predominantly governed by bile acid availability [4]. Unexpectedly, bile acids and FXR signaling have been reported to influence gut microbiota composition. Bile acids modulate gut microbial communities both directly and indirectly through immune responses and FXR signaling [62]. Interestingly, the microbial community also regulates bile acid metabolism and synthesis in an FXR-dependent manner [63]. For example, FXR-deficient mice are enriched with Desulfovibrionaceae, Deferribacteraceae, and Helicobacteraceae, which may be associated with the increased hepatic conjugated bile acids and lipids [64]. Furthermore, FXR is expressed in intestinal L cells and regulates GLP-1 secretion [65].

In addition to FXR, TGR5, another bile-acid-responsive receptor, plays a significant role in the regulation of lipid absorption [9]. Interestingly, gut microbiota can convert primary bile acids into secondary bile acids (e.g., LCA and DCA), which act as potent TGR5 agonists. Consequently, the composition and metabolic activity of gut microbiota influence the profiles and abundance of bile acids, thereby modulating TGR5 activation. Interestingly, TGR5-deficient mice exhibit a reduced bile acid pool, indicating that TGR5 also contributes to bile acid homeostasis [63]. In particular, activation of TGR5 signaling promotes intestinal GLP-1 secretion [66], which in turn regulates lipase secretion. Notably, depletion of gut microbiota increases fasting plasma GLP-1 levels while abolishing postprandial GLP-1 response [21]. Furthermore, compared with TGR5-knockout mice, ω-muricholic acid and hyocholic acid enhance GLP-1 secretion in wild-type mice [21]. These data indicate that gut microbiota is essential for maintaining postprandial GLP-1 response and that the bile acid–TGR5 signaling pathway, mediated by ω-muricholic acid and hyocholic acid, is involved in this process. Therefore, the interaction among targeted microbial communities, bile acids, and FXR and/or TGR5 signaling represents a promising strategy for regulating lipid absorption; however, further research is warranted.

Notably, bariatric surgery is currently the most effective intervention for severe obesity and its associated complications [67]. Alterations in bile acid homeostasis and changes in gut microbiota are considered key contributors to the metabolic improvements observed after surgery [68]. For instance, a meta-analysis reported that fasting bile acid levels—particularly those of 12α-hydroxylated bile acids—increase after Roux-en-Y gastric bypass (RYGB), whereas no significant changes are observed after sleeve gastrectomy (SG) [69]. Similarly, after bariatric surgery, fasting plasma total bile acid levels and 6α-hydroxylated bile acids (agonists of TGR5) increase, whereas fecal bile acid excretion decreases, suggesting enhanced bile acid reabsorption and activation of TGR5 [68]. Moreover, postoperatively, postprandial levels of fibroblast growth factor 19 rise, while the bile acid synthesis marker 7α-hydroxy-cholesten-4-one (C4) is suppressed, indicating activation of intestinal FXR [68]. However, vertical SG does not directly activate FXR in the liver or intestine; instead, it lowers intestinal bile acid levels, thereby reducing intestinal fat absorption [70]. These findings suggest that after bariatric surgery, the regulation of lipid absorption is not entirely dependent on FXR or TGR5 activation. Therefore, further investigation is warranted to elucidate the underlying mechanisms.

**Fig. 3.**
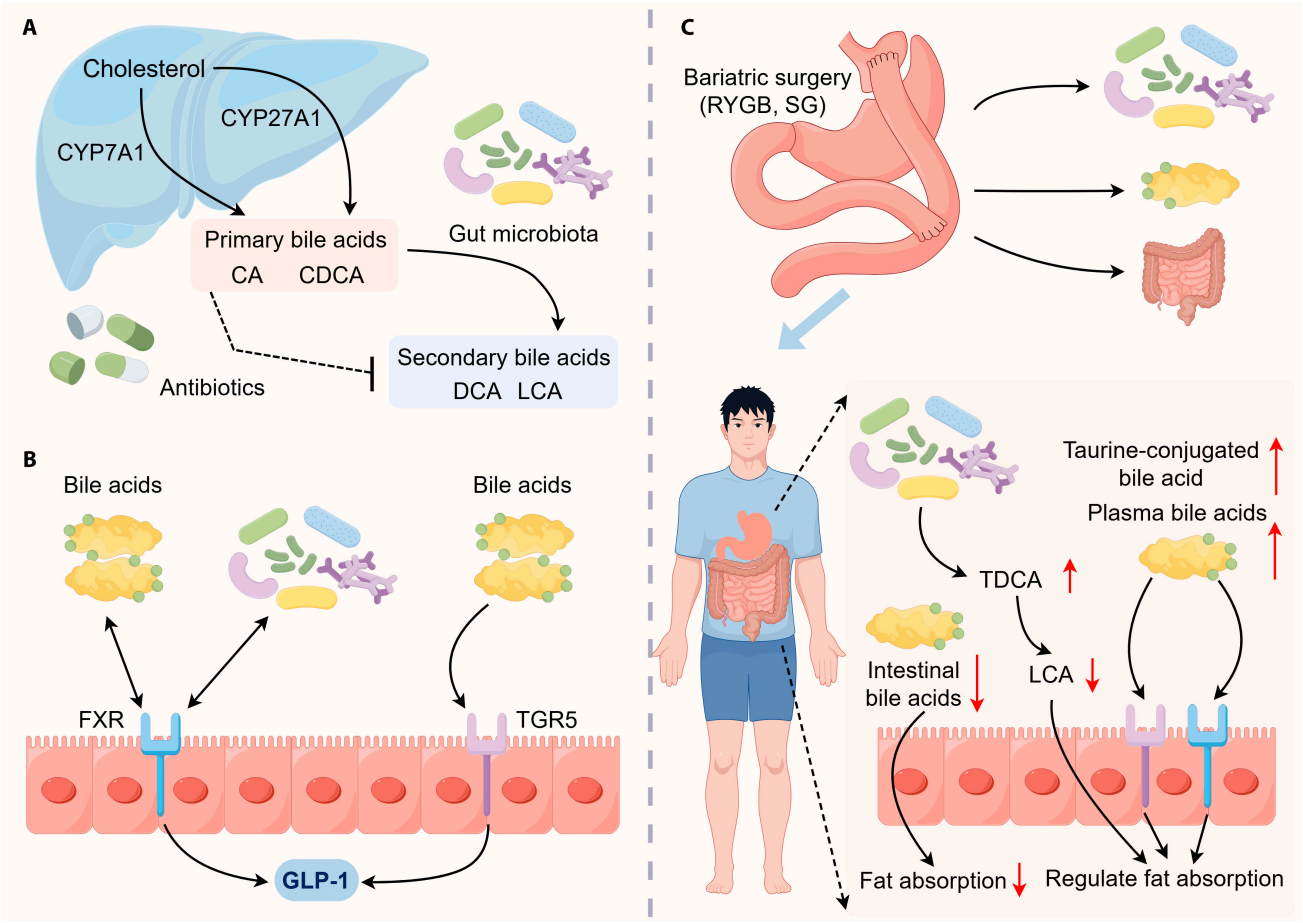
The bariatric surgery–gut microbiota–bile acid axis: potential mechanisms regulating lipid absorption. (A) The gut microbiota modulates the metabolism and synthesis of bile acids. (B) Interactions between the gut microbiota and bile acids activate FXR and TGR5, thereby stimulating GLP-1 secretion. (C) Bariatric surgery regulates lipid absorption by modulating gut microbiota and bile acids. CA, cholic acid; CDCA, chenodeoxycholic acid; DCA, deoxycholic acid; LCA, lithocholic acid; RYGB, Roux-en-Y gastric bypass; SG, sleeve gastrectomy; TDCA, taurodeoxycholic acid.

Notably, RYGB or repeated FMT from RYGB donors to diet-induced obese mice can induce alterations in gut microbiota [71]. Furthermore, transplantation of gut microbiota from mice that underwent RYGB into germ-free recipient mice, compared with recipients colonized with microbiota from sham surgery donors, resulted in a reduced body weight and a decreased fat mass [72]. These findings provide empirical support that post-RYGB alterations in the gut microbiota contribute to regulating lipid absorption and reducing host body weight and adiposity. Specifically, after bariatric surgery, gut-microbiota-mediated regulation of taurine metabolism increases the abundance of taurine-conjugated bile acid species in both the intestine and circulation. These bile acids activate intestinal FXR and systemic TGR5 signaling, thereby stimulating adaptive thermogenesis [71]. Interestingly, after SG, the levels of LCA (which can impair whole-body metabolism) in the gastrointestinal tract decreased. Unexpectedly, taurodeoxycholic acid, uniquely elevated in the small intestine after SG, is able to reduce LCA levels [73]. Overall, the bidirectional relationship between bile acids and gut microbiota, and the reciprocal effects of bariatric surgery and gut microbiota, may be central to elucidating the mechanisms through which the bariatric surgery–gut microbiota–bile acid axis regulates lipid absorption (Fig. 3).

### Interacting with gut immune cells

Microbial diversity and composition in the gut are highly associated with the number and fate of immune cells to affect host metabolism and immunity [74–76]. Among intestinal immune cells, T helper 17 (Th17) cells have been identified as directly involved in regulating lipid absorption [77]. High-fat diet consumption specifically reduces intestinal Th17 cell immunity. In contrast, the presence of Th17 cells confers protection against metabolic syndrome by decreasing intestinal lipid absorption, accompanied by the down-regulation of *CD36* expression in the intestine [77]. The mechanism is dependent on Th17-cell-derived interleukin-17 and occurs specifically in the distal intestine, but not in duodenum [77]. Interestingly, metabolites produced by gut microbiota, such as 3-oxolithocholic acid and isolithocholic acid, have the capacity to inhibit the differentiation of Th17 cells [78]. This indicates that the interplay between gut microbiota, bile acids, and Th17 cells may play a crucial role in regulating lipid absorption.

Notably, intestinal mucosal mast cells are also activated in response to dietary fat [79], which are reduced after antibiotic exposure, accompanying reduced lymphatic transport of TG and phospholipid [15]. B cells, macrophages, dendritic cells, and natural killer cells have also been reported to express lipid transporters, such as *CD36* [80,81]. Although the role of immune cells in lipid metabolism has been widely recognized [82,83], the effects and mechanisms of how these cells affect intestinal lipid absorption need further studies.

### Interacting with long noncoding RNA Snhg9

Long noncoding RNAs (lncRNAs) play crucial roles in various biological processes. Therefore, identifying potential lncRNAs associated with intestinal lipid absorption will enhance our understanding of the pathogenesis and provide insights into therapeutic strategies for metabolic diseases. Whole-transcriptome sequencing screens small nucleolar RNA host gene 9 (Sngh9) encoded lncRNAs as the most up-regulated transcript in germ-free mice, consistent with that in epithelial cells from antibiotic-treated mice [84]. These results confirm that healthy gut microbiota impede Snhg9. Although Sngh9 is widely studied in tumor biology [85,86], the effect of Sngh9 on intestinal lipid absorption is largely unknown. Recently, villin-Snhg9 transgenic mice have been generated and phenotypes are characterized by an improvement of diet-induced metabolic disorders, concomitant with less lipids in the intestine and more secretion in the feces through down-regulating *CD36* and *FABP4* [84,87]. Interestingly, bacteria that repress *Snhg9* expression have been identified, including *Salmonella enterica* serovar *Typhimurium* and segmented filamentous bacteria. This repression is mediated through myeloid cell–type 3 innate lymphoid cell signaling [84].

### Interacting with toll-like receptors

Toll-like receptors (TLRs), a class of pattern recognition receptors perceiving gut bacteria, are generally suppressed by healthy microbial compositions [88,89]. Dietary fat shapes gut microbiota and results in lipid deposition [90,91], along with activation of TLR2 and TLR4 [92]. Ligand-mediated stimulations of TLR2 and TLR4 in human peripheral blood mononuclear cells down-regulate *CD36* expression on the surface of monocytes [93]. The mechanisms may be associated with TLR-induced TNFα production as recombinant TNFα has been reported to inhibit *CD36* expression [93]. In addition, TLR4 is also involved in oxidized-lipid-mediated dipeptidyl peptidase 4 expression [94], which has been reported to regulate lipase secretion [24]. Serving as the downstream signal, Myd88 deficiency in T cells up-regulates intestinal genes involved in lipid absorption, including *Slc27a4* (encoding FABP4) and *CD36*, thereby increasing serum fatty acid levels while reducing the fatty acid content in the cecum [40].

### Interacting with intestinal permeability

Gut microbiota plays a crucial role in regulating intestinal permeability. Intestinal permeability is specifically regulated by tight junction proteins—including occludins, tricellulins, claudins, adhesion molecules, and zonula occludens—which form intercellular junctions between intestinal epithelial cells and selectively permit the passage of water and other small molecules into the circulation [37,95]. Notably, chylomicrons are transported across the basolateral membrane of intestinal epithelial cells via exocytosis but cannot directly traverse the intestinal basement membrane [95,96]. As a result, they temporarily accumulate in intercellular spaces. However, disrupted gut microbiota can increase intestinal permeability and widen intercellular spaces, thereby promoting the transport of chylomicrons.

Furthermore, following fat intake, the accumulation of chylomicrons in these spaces causes the basement membrane to bulge and temporarily rupture, thereby enabling the entry of chylomicrons into the lamina propria [95,96]. Interestingly, a high-fat diet is often associated with the disruption of intestinal microbiota, which further exacerbates the increase in intestinal permeability and provides a paracellular pathway for lipids to cross the basement membrane [95,96]. Notably, probiotics can promote the expression of tight junction proteins, increase mucus secretion, and enhance ethanolamine metabolism, thereby improving the health of intestinal epithelial cells while reducing intestinal permeability and lipid absorption through the production of butyrate [37,97,98].

## Microbial Manipulations of Intestinal Lipid Absorption for Lipid Metabolic Disorders

**Fig. 4.**
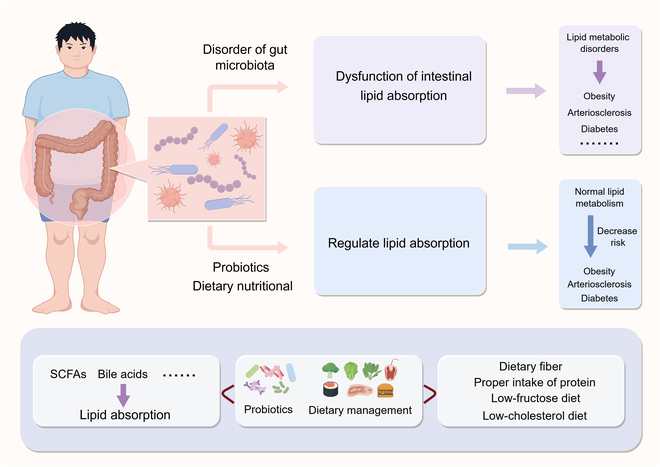
Probiotics and dietary interventions modulate lipid absorption through the regulation of gut microbiota.

Dysfunction of intestinal lipid absorption widely correlates with obesity, arteriosclerosis, and diabetes; thus, manipulations of gut microbiota targeting intestinal lipid absorption may serve as a novel method to treat or prevent metabolic disorders. Dietary composition and overall nutritional status are key determinants of gut microbiota structure and function [99], which helps scientists design a healthy dietary plan to govern metabolic diseases (Fig. 4).

### Probiotics

Given that probiotics directly improve gut microbial composition, there is growing interest in screening potential probiotic strains with specific effects on lipid absorption for preventive and therapeutic applications in metabolic disorders [100]. High-fat diet typically reduces the abundance of various probiotic bacteria, such as segmented filamentous bacteria, which could potentially be targeted to modulate lipid absorption and alleviate metabolic disorders. Notably, a rapid loss of segmented filamentous bacteria occurs in adults with metabolic syndrome, and colonization with segmented filamentous bacteria specifically decreases intestinal lipid uptake and absorption by controlling the epithelial expression of *CD36* [77]. The primary improvements in body mass index, lipid markers, insulin and glucose metabolism, inflammatory markers, and adiposity are from a large range of probiotics, including *Lactobacillus*, *Streptococcus*, and *Bifidobacterium* [101,102]. Indeed, these probiotics are widely reported to inhibit intestinal lipid absorption and exhibit a metabolic benefit [36,103]. Specifically, the metabolic products of probiotics—such as SCFAs, bile acids, and bile salt hydrolases—can markedly reduce lipid levels, and the underlying mechanisms have been thoroughly summarized in previous reviews [9,102,104].

However, lipid-absorption-promoting bacteria are also identified in the *Lactobacillus* genus. For example, *Lactobacillus murinus* gavage markedly increases intestinal lipid absorption and fatty acid uptake in germ-free mice [105]. In our previous study, *Lactobacillus johnsonii* and *Lactobacillus reuteri* are isolated from fatty pigs and exhibits a lipid-absorption-promoting effect by targeting lipid transporters [106]. These differences may be attributed to bacterial sources and genotypes; therefore, efficient probiotic screening targeting intestinal lipid absorption is necessary to inform diverse probiotic strategies.

### Dietary nutritional management

The interaction between diet and gut microbiota has been extensively reviewed, and leveraging dietary interventions to precisely engineer the gut microbiome represents a promising strategy for preventing metabolic diseases. For example, using the geometric framework model covering dietary energy, protein, fat, and carbohydrate factors, longevity and health are optimized in low-protein and high-carbohydrate diet [107]. Specifically, diets characterized by a low protein content (10%) and a high carbohydrate content (70%) yield the most favorable metabolic outcomes when the carbohydrate component primarily consists of resistant starch [108]. Conversely, the least favorable outcomes are observed when the carbohydrate component is a 50:50 mixture of the monosaccharide fructose and glucose [108]. Thus, dietary fiber offers a promising solution for metabolic disorders by modulating gut microbiota. In a randomized, placebo-controlled crossover trial, dietary supplementation with resistant starch enriches the gut populations of *Bifidobacterium adolescentis*, *Bifidobacterium longum*, and *Ruminococcus bromii*, leading to reduced lipid absorption in participants who are overweight or obese [36]. The antidiabetic effect of dietary fiber (konjac glucomannan) has also been demonstrated through the inhibition of lipid absorption and modulation of the gut microbiome in a rat model of type 2 diabetes mellitus [109].

A low-protein diet is recommended for various metabolic diseases due to the benefits on healthy lifespan, adiposity, and glucose tolerance [110]. However, refeeding a high-protein diet tends to inhibit intestinal lipid absorption and fat accumulation after dieting, which is generally accompanied by quick fat accumulation and an increase in intestinal *Lactobacillus* and its metabolites [105]. In addition, high-protein-diet-shaped gut microbiota produces a large number of extracellular vesicles, which further activates epithelial TLR4 activation [111], while the activation of TLR4 has been reported to down-regulate *CD36* expression [93]. In summary, targeting intestinal lipid absorption through a high-protein diet may be an effective strategy for preventing obesity after dieting; however, caution is advised when applying this diet under normal physiological conditions due to the opposite metabolic phenotypes.

Consumption of fructose, abundant in honey and fruits, is linked to the rising incidence of obesity and other metabolic diseases [112–114]. Dietary fructose increases intestinal villus length in the duodenum and proximal jejunum by 25% to 40%, correlating with enhanced lipid absorption and accelerated obesity through modulation of the gut microbiome [42,115]. In fructose-challenged mice, 42 bacterial genera are distinguished, with the majority showing correlations with obesity-related phenotypic variables, including body weight, perirenal fat, epididymal fat, and liver fat percentage [116]. In addition, fructose exposure triggers a bacterial stress response that promotes phage production, thereby influencing bacterial community composition [117]. Although altered viral compositions and weakened viral–bacterial correlations in the intestine are characterized in obesity and type 2 diabetes mellitus [118], the role of intestinal viruses in lipid absorption needs further studies.

Cholesterol, a major component of dietary lipids, is essential for maintaining cell membrane fluidity and for physiological processes such as the synthesis of steroid hormones and fat-soluble vitamins [119]. Notably, the average daily intake of cholesterol ranges from approximately 200 to 600 mg [119]. Clinical and experimental studies have demonstrated that a high-cholesterol diet can induce nonalcoholic fatty liver disease in both obese and nonobese individuals [120]. Specifically, a high-cholesterol diet induces dysbiosis of gut microbiota, characterized by reduced microbial diversity and decreased abundance of *Bifidobacterium* and *Bacteroides* [121]. Furthermore, a high-cholesterol diet increases the level of taurocholic acid while decreasing that of 3-hydroxyindolepropionic acid [121]. Notably, 3-hydroxyindolepropionic acid inhibits cholesterol-induced lipid accumulation and cell proliferation, whereas taurocholic acid exacerbates cholesterol-induced TG accumulation [121]. Therefore, appropriate control of dietary cholesterol intake and the selection of healthy foods are essential for maintaining human health. Moreover, the complex interactions between gut microbiota and cholesterol metabolism present new opportunities for future research and therapeutic interventions.

## Conclusions and Perspectives

Over the past several decades, significant progress has been made in elucidating the mechanisms underlying intestinal digestion and absorption of dietary lipids. The interaction between gut microbes and intestinal lipid absorption has increasingly been recognized as a critical area of study, particularly as a potential therapeutic target. In this review, we summarize the role of gut microbiota in mediating lipase secretion and lipid absorption, with a focus on lipid transporters, bile acids, immune cells, SNHG9, TLRs, and intestinal permeability. We also discuss microbial manipulations through probiotics and dietary interventions to regulate intestinal lipid absorption and thereby govern metabolic disorders. Although much has been learned about the interaction between gut microbiota and lipid absorption, a variety of concerns remain poorly understood. For example, the same bacterial species may exhibit opposite metabolic phenotypes. Indeed, *B. thetaiotaomicron*, a symbiotic bacterium in the human gut, exacerbates metabolic disorders by enhancing lipid digestion and absorption [38]. Conversely, gavage with *B. thetaiotaomicron*—enriched by intermittent fasting intervention—shows an inverse correlation with obesity and atherosclerotic cardiovascular diseases [122] and alleviates diet-induced adiposity [39]. Therefore, comprehensive and meticulous documentation of specific probiotic strains, including their genomic information and potential interactions with dietary nutrients or pharmaceuticals, is essential. Recently, gut microbial rhythms along with central and peripheral host circadian clock functions have been well-established with emphases on metabolic diseases [123–127], while the mechanisms by which circadian rhythms modulate gut microbiota and subsequently regulate intestinal lipid absorption remain to be elucidated. Gut fungi and viruses and their interactions with bacteria are expected to be involved in metabolic diseases [128]; it has remained a significant challenge to dissect fungal or viral characteristics in intestinal lipid absorption due to microbial complexity and interactions. Moreover, the role of low-molecular-weight compounds in nutrition in revealing the interactions between diet, gut microbiota, and lipid absorption also informs future diagnosis and therapy. Therefore, future research should further investigate the interaction between gut microbiota and lipid absorption across multiple dimensions, aiming to identify potential targets and develop microbial intervention strategies for metabolic disorders.

## Data Availability

Data sharing is not applicable to this article as no new data were created or analyzed in this study.
